# Enumerating tree-like chemical graphs with given upper and lower bounds on path frequencies

**DOI:** 10.1186/1471-2105-12-S14-S3

**Published:** 2011-12-14

**Authors:** Masaaki Shimizu, Hiroshi Nagamochi, Tatsuya Akutsu

**Affiliations:** 1 Graduate School of Informatics, Kyoto University, Yoshida, Kyoto 606-8501, Japan; 2 Bioinformatics Center, Institute for Chemical Research, Kyoto University, Gokasho, Uji, Kyoto 611-0011, Japan

## Abstract

**Background:**

Enumeration of chemical graphs satisfying given constraints is one of the fundamental problems in chemoinformatics and bioinformatics since it leads to a variety of useful applications including structure determination of novel chemical compounds and drug design.

**Results:**

In this paper, we consider the problem of enumerating all tree-like chemical graphs from a given set of feature vectors, which is specified by a pair of upper and lower feature vectors, where a feature vector represents the frequency of prescribed paths in a chemical compound to be constructed. This problem can be solved by applying the algorithm proposed by Ishida *et al*. to each single feature vector in the given set, but this method may take much computation time because in general there are many feature vectors in a given set. We propose a new exact branch-and-bound algorithm for the problem so that all the feature vectors in a given set are handled directly. Since we cannot use the bounding operation proposed by Ishida *et al*. due to upper and lower constraints, we introduce new bounding operations based on upper and lower feature vectors, a bond constraint, and a detachment condition.

**Conclusions:**

Our proposed algorithm is useful for enumerating tree-like chemical graphs with given upper and lower bounds on path frequencies.

## Introduction

Development of novel drugs is one of the major goals in chemoinformatics and bioinformatics. To achieve this purpose, it is important not only to investigate common chemical properties over chemical compounds having common structural patterns [1-3] but also to study methods of enumerating chemical structures satisfying given constraints. The enumeration of chemical structures has a long history. Actually, Cayley [4] considered the enumeration of structural isomers of alkanes in the 19th century. Applications for the enumeration of chemical compounds include structure determination using mass-spectrum and/or NMR-spectrum [5,6], virtual exploration of chemical universe [7,8], reconstruction of molecular structures from their signatures [9,10], and classification of chemical compounds [11].

In the field of machine learning, the *pre-image problem *[12,13] has been studied. In this problem, a desired object is computed as a feature vector in a feature space, and then the feature vector is mapped back to the input space, where this mapped back object is called a pre-image. The definition of the feature vectors based on the frequency of labeled paths [14,15] or small fragments [11,16] has been widely used. Akutsu and Fukagawa [17] formulated the graph pre-image problem as the problem of inferring graphs from the frequency of paths of labeled vertices, which corresponds to the pre-image problem, and proved that the problem is NP-hard even for planar graphs with bounded degrees [17]. Nagamochi [18] proved that a graph determined by frequency of paths with length 1 can be found in polynomial time if any.

To enumerate tree-like chemical graphs, Fujiwara *et al*. [19] proposed a branch-and-bound algorithm which consists of a branching procedure based on the tree enumeration algorithm due to Nakano and Uno [20,21] and bounding operations designed by the path frequency and the atom-atom bonds. In addition, to reduce the size of search trees, Ishida *et al*. [22] introduced a new bounding operation, called the *detachment-cut*, based on the result by Nagamochi [18]. Implementations of the algorithm proposed by Ishida *et al*. [22] are available at a web server (*http://sunflower.kuicr.kyoto-u.ac.jp/tools/enumol/*) for enumerating tree-like chemical graphs with given path frequency. However, an instance with constraint which is specified by one feature vector admits no solution in many cases. Therefore, it is needed to introduce a more relaxed constraint than a single feature vector to obtain some solutions in the tree-like chemical graph enumeration problem.

In this paper, we are given a set of feature vectors, which is specified by a pair of upper and lower feature vectors, and enumerate all tree-like chemical graphs satisfying one of the vectors. It seems that this can be done by simply applying the algorithm proposed by Ishida *et al*. to each single feature vector in the given set. However, this method will take much computation time because in general there are many feature vectors in a given set. We propose a new exact branch-and-bound algorithm for the problem so that all the feature vectors in a given set are handled directly.

## Methods

### Preliminaries and problem formulation

**Figure 1:**
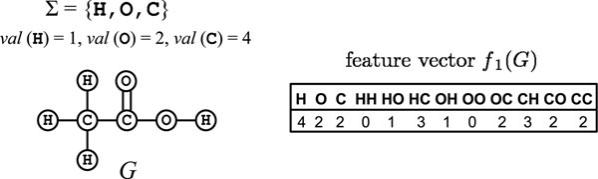
**A chemical compound and its feature vector**. An illustration of a (Σ, *val*)-labeled multitree *G* and its feature vector *f*_1_(*G*). Notice that multiple edges with the same end-vertices are treated as one edge, where *#OC* = *#CO* = 2.

A graph is called a *multigraph* if multiple edges (i.e., edges with the same end vertices) are allowed; otherwise it is called *simple*. A *path P* is a sequence *v*_0_, *e*_1_, *v*_1_, *e*_2_, *v*_2_, …, *e_k_*, *v_k_* of distinct vertices *v_i_* (*i* = 0, …, *k*) and edges *e_j_* that join *v*_*j* – 1_ and *v_j_* (*j* = 1, …, *k*). Without confusion we may write *P* = (*v*_0_, *v*_1_, …, *v_k_*). The length *|P|* of path *P* is defined to be *k*, i.e., the number of edges. Assume that a set Σ = {*ℓ*_1_,*ℓ*_2_, …,*ℓ_s_*} (i.e., chemical elements) is given. Let each label *ℓ* be associated with a valence *val*( *ℓ*) ∈ ℤ_+_. A multigraph *G* is called Σ-*labeled* if each vertex *v* has a label *ℓ*(*v*) ∈ Σ, and is called (Σ, *val*)*-labeled* if, in addition, the degree of each vertex *v* is *val*(*ℓ*(*v*)), i.e., the valence of the element *ℓ*(*v*). We regard chemical compounds as (Σ, *val* )-labeled, self-loopless, and connected multigraphs, where vertices and labels represent atoms and elements, respectively. For a path *P* = (*v*_0_, *v*_1_, …, *v_k_*), we call *ℓ*(*P*) = *ℓ*(*v*_0_), *ℓ*(*v*_1_), …, *ℓ*(*v_k_*) the *label sequence* of *P*. Given a label sequence *t*, let #*t* denote the number of paths *P* with *ℓ*(*P*) = *t* in a graph, where multiple edges with the same end-vertices are treated as a single edge and paths are considered to be “directed.” The *feature vector f_K_*(*G*) *of level K*(∈ ℤ_+_) of *G* is defined to be the vector whose entry *f_K_*(*G*)[*t*] (*|t|* ≤ *K*) represents #*t*. See Fig. 1 for an example.

Let *deg*(*v; G*) denote the degree of a vertex *v* in a graph *G*. The tree-like chemical graph enumeration problem with given one feature vector can be formulated as follows [19].

#### Enumeration of Tree-like chemical graphs with given Path Frequency (ETPF)

Given a set Σ of labels, a valence function *val* : Σ → ℤ_+_ and a feature vector *g* of level *K*, find all (Σ, *val*)-labeled multitrees *T* such that *f_K_*(*T*) = *g* and *deg*(*v;T*) = *val*(*ℓ*(*v*)) for all vertices *v* ∈ *V*(*T*).

Observe that a large number of chemical compounds contain a high proportion of hydrogens. Based on this fact, another model can be considered in the problem ETPF by removing all hydrogen atoms. These two different models were proposed by Fujiwara *et al*. [19] and Ishida [23].

**Figure 2:**
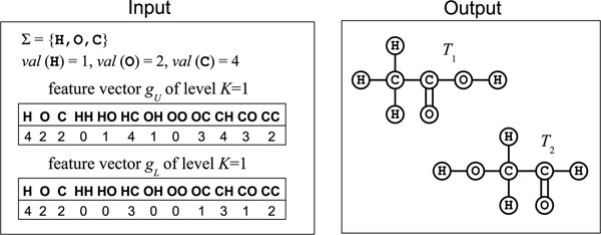
**An instance of ETULF.** An instance of ETULF with upper and lower feature vectors, which admits two different solutions.

In this paper, we consider the problem of enumerating all tree-like chemical graphs based on given upper and lower feature vectors because we want to relax the feature vector constraint in the problem ETPF. For feature vectors *g*_1_ and *g*_2_ of level *K*, we define *g*_1_ ≤ *g*_2_ to be *g*_1_[*t*] ≤ *g*_2_[*t*] for any label sequence *t* (|*t*| ≤ *K*). The problem of enumerating tree-like compounds from given two feature vectors can be formulated based on the problem ETPF as follows (see Fig. 2 for an illustration).

#### Enumeration of Tree-like chemical graphs with given Upper and Lower bounds on path Frequencies (ETULF)

Given a set Σ of labels, a valence function *val* : Σ → ℤ_+_ and feature vectors *g_U_* and *g_L_* of level *K* (*g_L_* ≤ *g_U_*), find all (Σ, *val*)-labeled multitrees *T* such that *g_L_* ≤ *f_K_*(*T*) ≤ *g_U_* and *deg*(*v;T*) = *val*(*ℓ*(*v*)) for all vertices *v* ∈ *V*(*T*).

For the problem ETULF, we assume that *g_L_*(*ℓ*) = *g_U_*(*ℓ*) for an atom type *ℓ* ∈ Σ, where *g*(*L*) denotes the entry in *g* that corresponds to a label sequence *L* (thus *g*(*ℓ*) specifies the number of vertices of label *ℓ*) and that *g_L_*(*L*) ≤ *g_U_*(*L*) for any label sequence *L* (*|L|* ≥ 2).

Note that the number *n* of vertices is given by Σ_*ℓ*∈Σ_*g*(*ℓ*). To solve the problem ETULF, we start with an empty graph, and repeatedly extend the current tree *T* by appending a new vertex with each label *ℓ* ∈ Σ to obtain a *valid* tree (a tree that does not violate any constraints on output trees) one by one until we get *n* vertices. In order to avoid duplicate outputs, we follow the branch-and-bound framework of Fujiwara *et al*. [19], which first defines a canonical representation for isomorphic trees, and then lists them using the algorithm of Nakano and Uno [20,21] (the branching operation) discarding invalid trees with some bounding operations. Since we cannot directly use the bounding operation proposed by Ishida *et al*. [22] due to upper and lower constraints, we introduce some new bounding operations.

### Canonical representation of trees and the branching operation

In this section, we explain a canonical representation of trees introduced by Fujiwara *et al*. [19] and the branching operation based on the canonical representation.

First of all, we introduce a root of a tree based on the following theorem.

**Theorem 1 (Jordan **[24]**) ***For any tree with n′ vertices*, either there exists a unique vertex v* such that each subtree obtained by removing v* contains at most*vertices*, or there exists a unique edge e* such that both of the subtrees obtained by removing e* contain exactly*vertices*.

Such a vertex v* and an edge e* in Theorem 1 are called *unicentroid* and *bicentroid*, respectively. Either unicentroid or bicentroid is called as *centroid*. Note that there exists a bicentroid only for an even *n′*. Since a case of bicentroid is similar to a case of unicentroid, now we only explain a case of unicentroid.

Next we introduce a canonical representation of trees that must be unique up to isomorphism. Let *T* be a tree of *n* vertices rooted at a vertex *v*_0_ (which is not necessarily its unicentroid). Suppose that it is embedded in the plane as an ordered tree, where *v*_0_ is located at the top part. Without loss of generality, let *v*_0_, *v*_1_, …, *v*_*n* – 1_ be indexed by the depth-first search (DFS) that starts from *v*_0_ and visits vertices from the left to the right. Define the *depth d*(*v*) of a vertex *v* to be the length of the (unique) path from *v*_0_ to *v* in *T*. The *depth-label sequence* of *T* (*L*(*T*)) is defined to be

Given an arbitrary order of labels, we define the order of depth-label sequences as follows. For any *T*_1_ and *T*_2_, we denote *L*(*T*_1_) >*L*(*T*_2_) if *L*(*T*_1_) is *lexicographically* larger than *L*(*T*_2_). Then the *canonical representation* of a rooted tree is defined by the *largest* depth-label sequence among all its plane embeddings. Actually this is equivalent to the *left-heavy* plane embedding [20,21].

Thus our branching task is to list all centroid-rooted left-heavy trees with *n* vertices and *m* (= |Σ|) labels. Following the scheme [20,21], we define a *parent-child* relation between two left-heavy trees. The *parent P*(*T*) of a left-heavy tree *T* is obtained from *T* by removing its *rightmost* leaf. Clearly *P*(*T*) is still left-heavy In this way, we can define a *family tree* of left-heavy trees whose leaves are exactly what we want to obtain.

Therefore we only need to enumerate the (leaf) nodes of . This can be done by starting from the empty tree (the root node of ) and repeatedly appending a new leaf to some appropriate place on the rightmost path of the current tree. Our branching operation employs the algorithm of Nakano and Uno [20,21], which extends the current tree *T* (i.e., finds a child of *T*) in *constant* time [19].

### Bounding operations

In this section, we explain how to check the validity of the current tree *T*. If we can conclude that *T* and all its descendants are not valid, then we can discard *T*. Our bounding operation discards *T* if at least one of the following criteria is violated:

**(C1)** The root of *T* remains the centroid of an output (the centroid constraint);

**(C2) ***deg*(*v;T*) ≤ *val*(*l*(*v*)) for all *v* ∈ *V*(*T*) (the valence constraint);

**(C3) ***f_K_*(*T*) ≤ *g_U_*, and *|T|* = *n* and *g_L_* ≤ *f_K_*(*T*) (the feature vector constraint);

**(C4) ***T* can be extended to a connected and loopless tree with *n* vertices (the detachment constraint);

**(C5) ***T* can have a descendant which has an appropriate number of multiple bonds (the multiplicity constraint).

(C1) and (C2) are the same as the work by Fujiwara *et al*. [19] and not difficult to check. (C3) and (C4) are different from the work by Fujiwara *et al*. [19] and Ishida *et al*. [22] due to upper and lower constraints. (C5) is a new bounding operation that we propose in this paper. In the following three subsections, we will discuss three bounding operations resulting from (C3), (C4), and (C5), called as *feature-vector-cut*, *detachment-cut*, and *multiplicity-cut*, respectively.

#### Feature-vector-cut procedure

In the problem ETULF, we cannot use the bounding operation proposed by Fujiwara *et al*. [19] directly due to upper and lower feature vectors, but we can introduce a bounding operation based on upper and lower feature vectors by modifying Fujiwara *et al*.’s work slightly.

Let *T* denote a current tree, *f_K_*(*T*) denote the feature vector of *T*, *g_u_* denote a given upper feature vector, and *g_L_* denote a given lower feature vector. By the feature vector constraints in the problem ETULF, we check the following condition.(1)

If *T* violates (1), then we discard *T*.

In addition, if |*T*| = *n*, then we check the following condition based on the constraint of upper and lower feature vectors.(2)

If *T* violates (2), then we discard *T*.

#### Detachment-cut procedure

This subsection describes the definition of detachment [18] and a new bounding operation based on it for the problem ETULF. Let *G* be a multigraph that may have self-loops, which represents the graph obtained from a chemical graph *H* by contracting the vertices with the same label into a single vertex, where each vertex in *G* corresponds a label in *H* (note that we do not eliminate any edges in *H* in contracting vertices to obtain *G*). A process of regaining *H* from *G* is described as follows. Given a function *r* : *V*(*G*) → ℤ_+_, an *r-detachment H* of *G* is a multigraph obtained from *G* by splitting each vertex *v* ∈ *V*(*G*) into a set of *r*(*v*) copies of *v*, denoted by *W_v_* = {*v*^1^, *v*^2^ …, *v^r^*^(^*^v^*^)^}, so that each edge {*u*, *v*} ∈ *E*(*G*) joins some vertices *u^i^* ∈ *W_u_* and *v^j^* ∈ *W_v_*. Hence an *r*-detachment *H* of *G* is not unique in general. A self-loop {*u*, *u*} in *G* may be mapped to a self-loop {*u^i^*,*u^i^*} or a non-loop edge {*u^i^*,*u^j^*} in a detachment *H* of *G*. Note that, for all vertex pairs {*u*, *v*} ∈ *V*(*G*), the number of edges between subsets *W_u_* and *W_v_* in *H* is equal to that of edges between vertices *u* and *v* in *G*.

**Figure 3:**
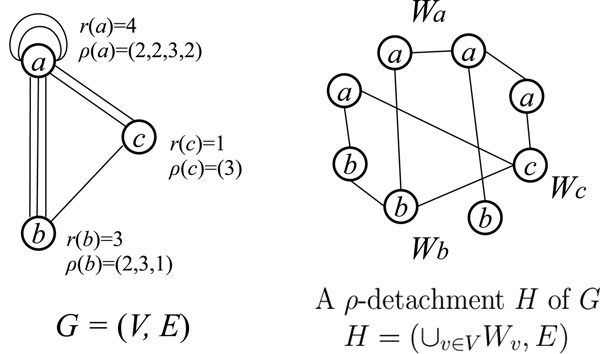
**A multigraph and a *ρ*-detachment**. A multigraph *G* and a *ρ*-detachment *H* of *G*.

To obtain a chemical graph *H* as an *r*-detachment *H* of *G*, we need to specify the degree of vertices (with the same label) in *H*. For a function *r* : *V*(*G*) → ℤ_+_, an *r-degree specification* is a set *ρ* of vectors  for *v* ∈ *V*(*G*) such that

which is necessary for all the edges incident to vertex *v* in *G* to be assigned to split vertices *v^i^* ∈ *W_v_* completely. An *r*-detachment *H* of *G* is called a *ρ-detachment* if each *v* ∈ *V* satisfies

which is a requirement that each vertex *v_i_* in *H* must have the prescribed degree . Figure 3 illustrates a *ρ*-detachment *H* for a graph *G* = (*V*, *E*) with *V* = {*a*, *b*, *c*}, a function *r* with *r*(*a*) = 4, *r*(*b*) = 3, *r*(*c*) = 1, and a degree specification *ρ* with *ρ*(*a*) = (2, 2, 3, 2), *ρ*(*b*) = (2, 3, 1), *ρ*(*c*) = (3). The next theorem gives a characterization of a multigraph *G* that admits a connected and loopless *ρ-*detachment.

**Theorem 2 (Nagamochi **[18]**) ***Let G* = (*V*, *E*) *be a multigraph*, *r* : *V* → ℤ_+_* and *. *Then G has a connected and loopless ρ-detachment H if and only if the following hold:*

*where r*(*X*) = Σ *_v_*_∈ _*_X_r*(*v*), *c*(*G′*) *denotes the number of connected components of a graph G′*, *G – X denotes the graph obtained from a graph G by removing the vertices in X together with all edges incident to vertices in X*, *and d*(*A*, *B; G*) *denotes the number of edges* (*u*, *v*) ∈ *E with u* ∈ *A and v* ∈ *B*.

Ishida *et al*. [22] proposed a bounding operation for the problem ETPF based on Theorem 2. However, we cannot use the bounding operation proposed by Ishida *et al*. for the problem ETULF due to upper and lower constraints. We now describe our new bounding operation based on detachments for the problem ETULF. The new bounding operation, called *detachment-cut* tests whether the current multitree *T* has a multitree that is consistent with given path frequencies among its descendants in the family tree, based on the difference between the feature vector *f_K_*(*T*) and the input feature vectors *g_U_* and *g_L_*.

Let *ℓ*_1_, *ℓ*_2_, …, *ℓ_s_* be input labels and *g_U_*, *g_L_* : Σ^≤ *K* + 1^ → ℤ_+_ be feature vectors. Let *r*_0_, …, *r_h_* be the vertices in the rightmost path to which a new leaf can be appended and  denote the number of vertices *r_j_* (0 ≤ *j* ≤ *h*) with *ℓ*(*r_j_*) = *ℓ_i_*. For each label sequence *t*, #*t* denotes the number of paths *P* in *T* with *ℓ*(*P*) = *t*. From *g_U_*, *g_L_*, and *T*, we define new feature vectors  and  of level *K* = 1 to be

**Figure 4:**
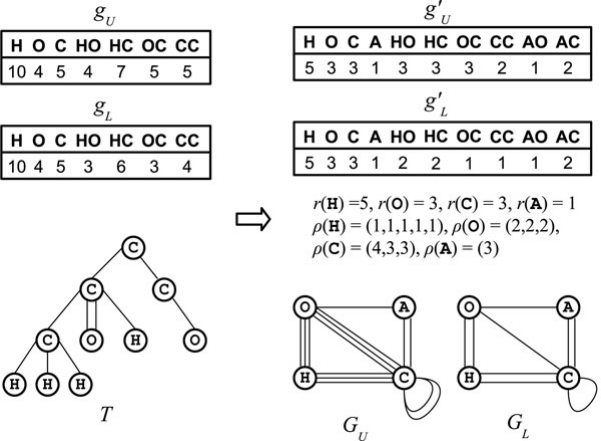
**Detachment-cut**. Bounding operation by detachment-cut, where vectors *g_U_*(*ℓ*, *ℓ′*), *g_L_*(*ℓ*, *ℓ′*), , and  are defined for unordered pairs {*ℓ*, *ℓ′*} and those with value=0 are omitted in the tables.

We next introduce a vertex with a new label *ℓ*_*s*+1_ of valence *h* + 1 (for example, label *A* in Fig. 4), a graph *G_U_* = (*V_U_*, *E_U_*) with a vertex set *V_U_* = {*v*_1_, …, *v_s_*, *v_s_*_+1_ | *ℓ*(*v_i_*) = *ℓ_i_*, 1 ≤ *i* ≤ *s* + 1} and edge set , and a graph *G_L_* = (*V_L_*,*E_L_*) with a vertex set *V_L_* = {*v*_1_, …, *v_s_*, *v_s_*_+1_ | *ℓ*(*v_i_*) = *ℓ_i_*, 1 ≤ *i* ≤ *s* + 1} and edge set . Note that *d*({*v_i_*}, {*v_j_*}; *G*) means a multiplicity of the edge {*v_i_*,*v_j_*} in a graph *G*. The function *r* and degree specification *ρ* are defined to be

Using *G_U_*, *G_L_*, *r*, and *ρ*, we can check if a current multitree *T* violates (C4). We need to check whether none of the following two conditions is violated.

(a) .

(b) *r*(*X*) + *c*(*G_U_ – X*) *– d*(*X*, *V_U_*; *G_U_*) ≤ 1 (∀*X* ⊆ *V_U_*, *X* ≠ ∅).

In the first condition, we check whether the number of the rest of bonds is large enough to satisfy the lower feature vector constraint. In the second condition, we check whether *T* has a connected and loopless descendant based on *G_U_* and Theorem 2.

#### Multiplicity-cut procedure

This subsection describes a new bounding operation based on multiplicity for the problem ETULF. Let *g*(*ℓ*) be the number of vertices with label *ℓ* ∈ Σ that are obtained from given the feature vector. Now we assume that *g*(*ℓ*) for all *ℓ* ∈ Σ are fixed in the problem ETULF. Then we can calculate the number of edges in output trees in the problem ETULF. Let *n* be the number of vertices in output trees. If we treat a multiple edge as a set of single edges, the number of edges *e_m_* in an output tree is given by:

On the other hand, if we treat a multiple edge as a simple one, the number of edges *e_s_* in an output tree is equal to *n –* 1 due to the tree-like constraint. Now we consider

which means that only *M* edges are used to construct multiple bonds in an output tree. Note that *M* ≥ 0. We calculate *M* from an input of the problem ETULF before the enumeration algorithm starts.

Let *T* = (*V*, *E*) be a multitree, and *m_e_* denote the multiplicity of *e* ∈ *E*. *The multiplicity M*(*T*) *of T* is defined to be

Now we describe the *multiplicity-cut* based on *M*(*T*) and *M*.

Let *T* be the current rooted multitree in the branching operation, *M*(*T*) be the multiplicity of *T*, *RP*(*T*) = (*r*_0_, *r*_1_, …, *r_k_*) be the rightmost path of *T*, *T_i_* be the new rooted multitree obtained by appending a new leaf *p* to a vertex *r_i_* (0 ≤ *i* ≤ *k*), and *RP*(*T_i_*) be the rightmost path of *T_i_* . The rightmost path *RP*(*T_i_*) of *T_i_* is updated by appending *p* to the end of *RP*(*T*) when a new leaf *p* is appended to *r_i_*, that is, *RP*(*T_i_*) = (*r*_0_, *r*_1_,…, *r_i_*, *p*). Then we can determine the multiplicities of the edges {(*r_j_*, *r*_*j* – 1_), *j* = *k*, *k –* 1, …, *i* + 1} due to the valence constraint, at the same time, we update *M*(*T_i_*). We denote the multiplicity of an edge (*r_j_*, *r*_*j* – 1_) in *T_i_* by *Mul*(*r_j_*, *r*_*j* – 1_ | *T_i_*). When we update the multiplicity of the edge (*r_j_*,*r*_*j* – 1_), *M*(*T_i_*) is updated as follows:

By the definition of *M*, a valid multitree *T_i_* satisfies(3)

**Figure 5:**
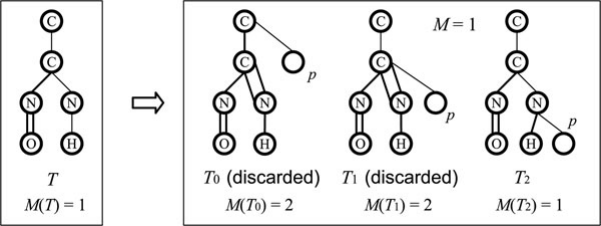
**Multiplicity-cut**. An illustration of the multiplicity-cut procedure, where *M* = 1.

If *T_i_* violates (3), then we discard *T_i_* . See Fig. 5 for an illustration of this.

## Results

This section reports the experimental results of our algorithm. First of all, we mention that the problem ETULF can be solved by applying the algorithm proposed by Ishida *et al*. [22] to each single feature vector in a given set of feature vectors, i.e., the problem ETULF can regard as a set of the problem ETPF. Then we call an algorithm for the problem ETULF based on the algorithm proposed by Ishida *et al*. RepEnum (Repeated Enumeration). On the other hand, we call our algorithm SimEnum (Simultaneous Enumeration). It is to be noted that RepEnum is one of the fastest tools to enumerate tree-like chemical structures from a given molecular formula (i.e., feature vector with *K* = 0) [22] and, to our knowledge, there does not exist any other available tool to enumerate chemical structures from a given feature vector based on path frequency (i.e., feature vector with general *K*).

Now we compare the performances of two algorithms, SimEnum and RepEnum, and we also compare the performances of two algorithms, SimEnum including multiplicity-cut and SimEnum not including multiplicity-cut. We have tested the algorithm SimEnum for some widths between upper and lower feature vectors. Tests were carried out on a PC with CPU AMD Athlon Dual Core Processor 5050e using instances based on some chemical compounds selected from the KEGG LIGAND database [25] (*http://www.genome.jp/ligand/*). Note that we treat a benzene ring contained in these compounds as a new virtual atom of valence six.

We define *w* ∈ ℤ_+_ to be a *width* between upper and lower feature vectors. From a feature vector *g*, we construct two feature vectors *g_U_* and *g_L_* as follows. For each entry *a* > 0 of *g*, let *g_U_* be the upper feature vector, where each entry *a_U_* is given by *a* + *w* and *g_L_* be the lower one, where each entry *a_L_* is given by max{0, *a – w*}. Note that if *w* = 0, then an instance for the problem ETULF is equivalent for the problem ETPF.

Table 1 and Additional file 1 show the results of the comparison. We find that the algorithm RepEnum cannot solve all the problems with *K* = 2 within the time limit since the number of feature vectors in a given set is exponentially increasing with *K*. On the other hand, Table 1 shows that the algorithm SimEnum can solve the problem much faster for a larger *K*. This shows that the algorithm SimEnum runs significantly faster than the algorithm RepEnum. It is also seen that RepEnum can only examine a very small portion of feature vectors in most cases. Additional file 1 shows that the algorithm SimEnum including multiplicity-cut runs faster than the algorithm SimEnum not including multiplicity-cut for almost all of the instances. This shows that the multiplicity-cut operation works well to improve enumeration efficiency.

**Table 1:** Comparison of previous method and our method

Entry Formula					SimEnum			RepEnum			
					
	*n*	*K*	*w*	*f_v_*	time (s)	nodes	solutions	time (s)	nodes	solutions	solved
		1	1	3^6^	1037.04	177,074,686	414,890	163.32	44,340,488	414,890	729
		2	1	3^18^	2.97	392,246	44	T.O.	2,381,360,000	N.F.	65,909,572
		3	1	3^34^	1.22	145,213	2	T.O.	3,293,260,000	N.F.	96,860,588
C00062	26	4	1	3^53^	0.33	34,539	1	T.O.	2,780,050,000	N.F.	81,766,176
C_6_H_14_N_2_O_4_		5	1	3^71^	0.24	20,361	1	T.O.	1,561,230,000	N.F.	45,918,529
		6	1	3^85^	0.25	15,166	1	T.O.	569,590,000	N.F.	16,752,647
		7	1	3^96^	0.18	14,547	1	T.O.	79,870,000	N.F.	2,349,117

		1	1	3^6^	T.O.	377,260,000	N.F.	T.O.	413,000,000	N.F.	460
		2	1	3^18^	7.24	845,760	25	T.O.	1,442,760,000	N.F.	70,175,902
		3	1	3^31^	2.81	307,151	7	T.O.	3,316,970,000	N.F.	195,115,882
C03343	37	4	1	3^47^	1.03	99,945	1	T.O.	2,494,780,000	N.F.	146,751,764
C_16_H_22_O_4_		5	1	3^64^	0.98	87,600	1	T.O.	1,050,480,000	N.F.	61,792,941
		6	1	3^82^	0.76	60,194	1	T.O.	315,820,000	N.F.	18,577,647
		7	1	3^99^	0.57	42,538	1	T.O.	41,450,000	N.F.	2,438,235

		1	1	3^8^	T.O.	157,320,000	N.F.	T.O.	200,490,000	N.F.	1,388
		2	1	3^26^	37.59	1,940,295	238	T.O.	2,911,390,000	N.F.	66,167,954
		3	1	3^48^	1.71	60,792	3	T.O.	2,673,940,000	N.F.	60,771,363
C07178	46	4	1	3^71^	0.35	14,248	1	T.O.	1,925,490,000	N.F.	43,761,136
C_21_H_28_N_2_O_5_		5	1	3^92^	0.27	10,866	1	T.O.	743,940,000	N.F.	16,907,727
		6	1	3^110^	0.27	10,680	1	T.O.	93,880,000	N.F.	2,133,636
		7	1	3^125^	0.24	9,276	1	T.O.	19,270,000	N.F.	437,954

		1	1	3^5^	T.O.	382,470,000	N.F.	T.O.	552,290,000	N.F.	61
		2	1	3^16^	T.O.	211,800,000	N.F.	T.O.	530,930,000	N.F.	10,451,912
		3	1	3^27^	1395.13	144,244,042	206	T.O.	3,314,260,000	N.F.	194,956,470
C03690	61	4	1	3^41^	121.36	11,332,363	4	T.O.	2,392,530,000	N.F.	140,737,058
C_24_H_38_O_4_		5	1	3^57^	83.70	6,978,557	2	T.O.	958,650,000	N.F.	56,391,176
		6	1	3^75^	40.11	2,923,819	1	T.O.	298,600,000	N.F.	17,564,705
		7	1	3^92^	16.50	1,096,128	1	T.O.	38,670,000	N.F.	2,274,705

Comparison of SimEnum and RepEnum for the problem ETULF.

Note:

(1) C00062, C03343, C07178, and C03630 are the chemical compounds in the KEGG LIGAND database, respectively;

(2) *n* is the number of vertices in an instance preprocessed by replacing each benzene ring with a new atom having six valences;

(3) *K* is the level of given feature vectors;

(4) *w* is the width for constructing upper and lower feature vectors;

(5) *f_v_* is the number of feature vectors in a given set;

(6) “time (s)” is the CPU time in seconds;

(7) T.O. means “time over” (the time limit is set to be 1,800 seconds);

(8) “nodes” is (the sum of) the number of nodes of family trees that are traversed;

(9) “solutions” is the number of all possible solutions;

(10) “solved” is the number of feature vectors which the algorithm RepEnum solved in the time limit; and (11) N.F. means “not found.”

Table 2 shows the results on the performance for varying width *w* for the problem ETULF. The search space in the problem ETULF is exponentially increasing with *w*. However, it seems that the number of search nodes and computation time are not exponentially increasing with *w*. This suggests that the algorithm SimEnum works efficiently for the large search space in the problem ETULF.

**Table 2:** Comparison of varying width

Entry Formula				SimEnum		
				
	*n*	*K*	*w*	time (s)	nodes	solutions
		2	0	0.51	55,196	6
		2	1	3.58	400,501	44
		2	2	7.58	835,509	503
C00062	26	2	3	10.84	1,163,548	2,351
C_6_H_14_N_2_O_4_		2	4	12.55	1,349,057	5,430
		2	5	13.29	1,431,075	9,852
		2	50	14.31	1,537,496	25,425

		2	0	0.34	35,952	9
		2	1	8.39	845,760	25
		2	2	48.27	4,815,369	41
C03343	37	2	3	149.83	14,781,738	305
C_16_H_22_O_4_		2	4	377.01	37,435,878	40,732
		2	5	639.68	63,459,180	106,870
		2	50	1118.75	110,703,034	510,079

		2	0	2.33	111,781	16
		2	1	46.81	2,246,578	238
		2	2	96.52	4,715,072	1,375
C07178	46	2	3	152.18	7,420,060	6,824
C_21_H_28_N_2_O_5_		2	4	179.42	8,744,563	19,180
		2	5	199.66	9,677,513	29,891
		2	50	255.01	12,292,587	54,861

		5	0	19.50	1,482,017	2
		5	1	220.14	16,063,569	5
		5	2	439.12	33,037,741	32
C03690	61	5	3	684.88	52,207,745	178
C_24_H_38_O_4_		5	4	1024.96	78,509,554	349
		5	5	1285.55	98,762,291	615
		5	50	T.O.	136,835,134	N.F.

Comparison of the performance for varying *w* for the problem ETULF.

Here, we briefly discuss practical values on *K* and *w* though we do not have concrete evidence and these values depend on target classes of chemical compounds. It is suggested from the results on similar feature vectors [9,10,15] that *K* between 3 to 10 should be used. Though there is no previous result on *w*, it is seen from Table 2 that *w* cannot be large because there may exist too many solutions. Therefore, *w* less than 4 should be used.

## Conclusions

We considered the problem of enumerating all tree-like chemical graphs from a given set of feature vectors, which is specified by upper and lower feature vectors based on frequencies of paths, and proposed a new exact branch-and-bound algorithm. Our experimental results show that our algorithm outperforms the naive algorithm based on a previous method. In comparison to the algorithm based on Ishida *et al*. [22], our algorithm can greatly reduce the number of search nodes and the computation time and enumerate all the feasible solutions in many instances.

However, the search space of the problem ETULF is much larger than that of the problem ETPF due to upper and lower constraints and in fact there are many search nodes for solving the problem ETULF by our algorithm. One of the future works is to improve the bounding operations, or introduce a new bounding operation. Actually, in the feature-vector-cut mentioned in subsection , information of a lower feature vector *g_L_* is only used if *|T|* = *n*. Another future work is to develop a web server that implements our proposed algorithm. Generalization of the proposed techniques for other types of kernel functions and other problems is also left as a future work.

## Competing interests

The authors declare that they have no competing interests.

## Author’s contributions

HN gave the basic idea based on discussions with TA and MS. MS developed and implemented the algorithms, and carried out the experiments. MS, HN, and TA authored and approved the manuscript.

## Supplementary Material

Additional file 1 **Comparison of multiplicity-cut** Comparison of SimEnum including multiplicity-cut and SimEnum not including multiplicity-cut for the problem ETULF. Note: (1) “add multiplicity-cut” is the algorithm SimEnum including multiplicity-cut; and (2) “no multiplicity-cut” is the algorithm SimEnum not including multiplicity-cut. Click here for file
