# Supplementary material for: Enumerating tree-like chemical graphs with given upper and lower bounds on path frequencies
Source: BMC Bioinformatics. 2011 Dec 14;12(Suppl 14):S3. doi: 10.1186/1471-2105-12-S14-S3 (PMC3287468; doi:10.1186/1471-2105-12-S14-S3)
Supplement: Additional file 1 — Comparison of multiplicity-cut Comparison of SimEnum including multiplicity-cut and SimEnum not including multiplicity-cut for the problem ETULF. Note: (1) “add multiplicity-cut” is the algorithm SimEnum including multiplicity-cut; and (2) “no multiplicity-cut” is the algorithm SimEnum not including multiplicity-cut. [file 1471-2105-12-S14-S3-S1.pdf]

Entry Formula	n	K	w	add multiplicity-cut time (s)	nodes	solutions	no multiplicity-cut time (s)	nodes	solutions
		1	1	1037.04	177,074,686	414,890	1055.98	201,771,484	414,890
		2	1	2.97	392,246	44	3.28	634,605	44
		3	1	1.22	145,213	2	1.46	242,620	2
C00062	26	4	1	0.33	34,539	1	0.44	56,603	1
$C_6H_{14}N_2O_4$		5	1	0.24	20,361	1	0.31	31,024	1
		6	1	0.25	15,166	1	0.22	21,771	1
		7	1	0.18	14,547	1	0.23	20,794	1
		1	1	T.O.	377,260,000	N.F.	T.O.	468,600,000	N.F.
		2	1	7.24	845,760	25	7.90	1,242,128	25
		3	1	2.81	307,151	7	3.26	484,467	7
C03343	37	4	1	1.03	99,945	1	1.27	167,956	1
$C_{16}H_{22}O_4$		5	1	0.98	87,600	1	1.30	146,015	1
		6	1	0.76	60,194	1	0.98	97,989	1
		7	1	0.57	42,538	1	0.72	63,775	1
		1	1	T.O.	157,320,000	N.F.	T.O.	118,262,003	N.F.
		2	1	37.59	1,940,295	238	39.52	3,172,578	238
		3	1	1.71	60,792	3	1.46	110,795	3
C07178	46	4	1	0.35	14,248	1	0.38	23,102	1
$C_{21}H_{28}N_2O_5$		5	1	0.27	10,866	1	0.34	17,648	1
		6	1	0.27	10,680	1	0.35	17,444	1
		7	1	0.24	9,276	1	0.30	14,728	1
		1	1	T.O.	382,470,000	N.F.	T.O.	622,630,000	N.F.
		2	1	T.O.	211,800,000	N.F.	T.O.	318,780,000	N.F.
		3	1	1395.13	144,244,042	206	T.O.	281,850,000	N.F.
C03690	61	4	1	121.36	11,332,363	4	413.98	54,157,242	4
$C_{24}H_{38}O_4$		5	1	83.70	6,978,557	2	363.72	39,070,732	2
		6	1	40.11	2,923,819	1	194.47	17,095,892	1
		7	1	16.50	1,096,128	1	77.59	5,666,412	1
